# Associations between polymorphisms in coagulation-related genes and venous thromboembolism: A meta-analysis with trial sequential analysis: Erratum

**DOI:** 10.1097/MD.0000000000007233

**Published:** 2017-06-08

In the article “Associations between polymorphisms in coagulation-related genes and venous thromboembolism: A meta-analysis with trial sequential analysis”,^[1]^ which appeared in Volume 96, Issue 13 in *Medicine*, “JJ, KL, and JZY contributed equally to this work” should have appeared as “JJ, KL, and JJZ contributed equally to this work.”
